# Neonatal hyperinsulinism in transient and classical forms of tyrosinemia

**DOI:** 10.1186/s13023-020-01642-y

**Published:** 2021-04-28

**Authors:** Swathi Sethuram, Mark A. Sperling, Jasmine Gujral, Christopher J. Romero

**Affiliations:** 1 Division of Pediatric Endocrinology and Diabetes, Department of Pediatrics, Icahn School of Medicine at Mount Sinai, 1 Gustave L. Levy Place, Box 1616, New York, NY 10029 USA; 2 Division of Pediatric Endocrinology, Yale School of Medicine, New Haven, CT USA

**Keywords:** Hyperinsulinism, Hypoglycemia, Hyperinsulinism in hereditary tyrosinemia I, Transient tyrosinemia of the newborn, Amino acids and hyperinsulinism, Hyperinsulinemic hypoglycemia

## Abstract

**Background:**

The spectrum of disorders associated with hyperinsulinemic hypoglycemia (HHI) has vastly increased over the past 20 years with identification of molecular, metabolic and cellular pathways involved in the regulation of insulin secretion and its actions. Hereditary tyrosinemia (HT1) is a rare metabolic disorder associated with accumulation of toxic metabolites of the tyrosine pathway due to a genetically mediated enzyme defect of fumarylacetoacetate hydrolase. Transient tyrosinemia of the newborn (TTN) is a benign condition with a maturational defect of the enzymes associated with tyrosine metabolism without any genetic abnormalities.

**Results:**

We describe two rare cases of HHI, one in a patient with HT1 and for the first time, in a patient with TTN. Each of our patients presented in the neonatal period with persistent hypoglycemia that on biochemical evaluation was consistent with HHI. Each patient received diazoxide therapy for 3.5 months and 17 months of life, respectively and HHI resolved thereafter.

**Conclusion:**

Despite the fact that HHI has been described in HT1 for several decades, no specific mechanism has been delineated. Although we considered the common embryonal origin of the liver and pancreas with the hepatotoxic effect in HT1 also impacting the latter, this was not a possible explanation for TTN. The commonality between our two patients is the accumulation of certain amino acids which are known to be insulinotropic. We therefore hypothesize that the excess of amino acids such as leucine, lysine, valine and isoleucine in our patients resulted in HHI, which was transient. Both patients responded to diazoxide. This novel presentation in TTN and the reassuring response in both HT1 and TTN to diazoxide will be useful to inform physicians about managing HHI in these patients. Further studies are required to delineate the mechanism of HHI in these infants.

## Background

Our knowledge of the metabolic and molecular basis of hyperinsulinemic hypoglycemia of infancy (HHI) has dramatically increased over the past 20 years [5, 6, 9, 18]. Monogenic forms of HHI have been identified with defects in the pancreatic ATP sensitive potassium (KATP) channels, transcription factors and enzymes of the insulin secretory pathway resulting in excessive production and secretion. Syndromic forms of HHI form a separate entity of genetic HHI disorders, which include Beckwith Wiedemann Syndrome, Kabuki Syndrome, Turner Syndrome and Hereditary Tyrosinemia Type I (de Leon and Stanley 2017) [10, 18].

Hereditary Tyrosinemia Type 1 (HT1, OMIM # 276700) is a rare autosomal recessive disorder with an estimated incidence of 1:100,000 [4]. HT1 results from an inability to metabolize the amino acid tyrosine due to deficiency of the enzyme fumarylacetoacetate hydrolase (FAH, EC number EC Number: 3.7.1.2) encoded by the *FAH* gene (Gene ID: 2184) on chromosome 15q25.1. This leads to the accumulation of succinylacetone, a toxic metabolite, resulting in damage to the liver, kidneys and peripheral nerves. HT1 has a broad spectrum of clinical presentations, which may be complicated by liver and kidney dysfunction if not appropriately treated [3, 15].

In contrast to HT1, transient tyrosinemia of the newborn (ORPHA:3402) is considered to be a benign condition caused by a maturational delay in the enzymatic action of the tyrosine catabolic process without any genetic mutation. This condition resolves spontaneously after several months of life and generally is without long-term sequelae [22].

HHI has rarely been reported in infants with HT1, but to date no cases have been reported in patients with transient tyrosinemia of infancy [1, 3] .We describe two unrelated patients with HHI in infancy, one with genetic and the other with transient forms of tyrosinemia. To maintain euglycemia, both patients initially required diazoxide, which was eventually discontinued in each of them.

We hypothesize that the metabolic derangements associated with tyrosinemia place a patient at risk for hyperinsulinism that may require pharmacological treatment. With scarce literature available on the subject, we explore some possible mechanisms for the etiology of this presentation in both these forms of tyrosinemia. This report should alert clinicians to the possible risk for hypoglycemia and its resolution with diazoxide treatment in patients with tyrosinemia, and highlights the need for further mechanistic studies.

## Case presentations

### Patient 1

This is a female infant born appropriate for gestational age [birth weight: 2.6 kg (Z = − 0.71 SD), birth length: 50 cm (Z = 0.78 SD)] at 37 weeks to a healthy mother [Gravida (G) 5,Parity (P)2], with no significant prenatal history. Physical examination at birth was unremarkable. The patient developed hypoglycemia at 2.5 h of life, with a point of care (POC) glucose measurement of 0.72 mmol/L (13 mg/dL). The patient required a combined glucose infusion rate (GIR) of up to 12 mg/kg/min [intravenous (IV) dextrose plus oral feeds] to maintain euglycemia. A critical sample was drawn when the plasma glucose was 1.9 mmol/L (34 mg/dL) on day of life 5 to evaluate the corresponding insulin and counter-regulatory hormone response (Table 1). Hyperinsulinism was diagnosed based on the elevated serum insulin (30.6 pmol/L or 4.4uU/ml) and C-peptide level (0.004 nmol/L or 1.1 ng/mL) with undetectable serum ketone levels (Table 1). Normal growth hormone and cortisol levels ruled out pituitary or adrenal dysfunction. The newborn screening test (drawn on day of life 3 and 22) reported elevated tyrosine levels in blood, but urine succinylacetone was negative, indicating that this was not HT1. The consulting genetic-metabolic team considered this to be transient tyrosinemia of infancy. Despite being on ad lib oral feeds with fortified (24 kcal/30 mL) formula, the patient continued to have intermittent hypoglycemic episodes (less than 3.3 mmol/L or 60 mg/dL beyond day 3 of life). Hence, diazoxide was started at two weeks of life at a dose of 12 mg/kg/day along with hydrochlorothiazide at 1 mg/kg/day. Thereafter, no further hypoglycemia or treatment-induced hyperglycemia was recorded. Given consistently normal blood glucose, the same diazoxide dose was continued during subsequent follow up visits, resulting in a lower per kilogram body weight dosing. The parents discontinued her medications at 3.5 months of life, while continuing to monitor her glucose levels via a POC glucose meter. She has since remained euglycemic with appropriate growth and development for age. Her transient tyrosinemia resolved biochemically when tested at 6 months of life and she was subsequently discharged from the endocrine clinic at 7 months of life.

**Table 1:** Patient profiles during hypoglycemia

	Patient 1	Patient 2
Laboratory samples (values consistent with hyperinsulinemic hypoglycemia) [7, 13]	Transient tyrosinemia of infancy	Hereditary tyrosinemia type I
Preserved plasma glucose < 3.3 mmol/L (< 60 mg/dL)	1.9 (34)	2.2 (39)
Beta-hydroxy butyrate < 1.8 mmol/L	< 0.2	< 0.2
C-peptide > 0.002 nmol/L (> 0.5 ng/dL)	0.004 (1.1)	0.007 (2.1)
Insulin > 13.9 pmol/L (> 2 uU/mL)	30.6 (4.4)	20.8 (3)
Growth hormone > 7.5 ug/L (> 7.5 ng/mL)	17 (17)	7.5 (7.5)
Cortisol 496 nmol/L (> 18 mcg/dL)	551.8 (20)	689.7 (25)

### Patient 2

This is a male infant born appropriate for gestational age [birth weight: 3.68 kg (Z = 1.24 SD), birth length: 52.1 cm (Z = 1.24 SD]) at 38 weeks to a G1P1 mother who developed gestational diabetes requiring insulin therapy from the 8^th^ month of pregnancy. At birth, physical examination was normal with no signs of dysmorphia. The patient was born at a regional hospital where he required IV fluids for hypoglycemia which was attributed to him being an infant of a diabetic mother. However, his hypoglycemia persisted with repeated POC glucose readings as low as 0.72 mmol/L (13 mg/dL). He received intravenous dextrose infusion in addition to oral feeds, requiring a combined GIR of up to 13.6 mg/kg/min to maintain euglycemia. His newborn screening test (drawn on day of life 2) was positive for elevated levels of succinylacetone, suggestive of HT1. The diagnosis of HT1 was confirmed by identifying a homozygous pathogenic mutation, c.192G > T, p.Q64H in the *FAH* gene. He was treated with nitisinone (2-[2-nitro-4-trifluoromethylbenzoyl]-1,3-cyclohexanedione, NTBC) and a tyrosine-free formula from day 9 of life. His tyrosine levels did normalize with this therapy by day 12 of life. However, he continued to have recurrent hypoglycemic episodes after two weeks of life. To establish a diagnosis for his hypoglycemia, a critical sample was drawn when his plasma glucose level was 2.2 mmol/L (39 mg/dL). Undetectable β-OH butyrate (< 0.2 mmol/L) with elevated C-peptide (0.007 nmol/L or 2.1 ng/mL) and insulin levels (20.8 pmol/L or 3 uU/mL) in this sample (Table I1 led to the diagnosis of hyperinsulinism. Serum growth hormone and cortisol showed an appropriate response to the hypoglycemia indicative of normal pituitary and adrenal function (Table 1). He was started on treatment with diazoxide at 10 mg/kg/day at two weeks of life, which stabilized glycemic control without additional need for intravenous glucose. Following discharge from the neonatal intensive care unit, the baby had moderate control of his tyrosinemia with nitisinone and dietary management. He did not have any further hypoglycemic episodes while on diazoxide. The patient gained adequate weight in the first year of life with appropriate development for age. Diazoxide dose was gradually weaned to sub-therapeutic levels (1.5 mg/kg/day) and discontinued at 17 months of life. The patient had one episode of hypoglycemia during an upper respiratory tract illness after stopping therapy. Diazoxide has since then not been restarted and the baby remains euglycemic to date.

## Discussion

We report two cases of neonatal hyperinsulinemic hypoglycemia in the setting of tyrosinemia; one case with hereditary tyrosinemia type I, which has been previously reported and another case with transient tyrosinemia of the newborn, which to our knowledge has not been described.

Despite similar nomenclature, Hereditary Tyrosinemia Type I and transient tyrosinemia of infancy have very different pathophysiology and prognosis. While the former is a permanent, genetic condition with significant morbidity and mortality, the latter is a benign and transient immaturity of enzyme(s). The common factor in both conditions, however, is the accumulation of tyrosine.

Hypoglycemia may affect up to 10% of healthy term infants in the first 24–48 h of life, but typically resolves spontaneously by day three of life. Screening and treatment of hypoglycemia is important to prevent adverse neurological sequelae. There remains some debate over the definition of hypoglycemia in the neonatal period and the glucose levels at which neurological development may be affected. More recently, the Pediatric Endocrine Society recommended treatment in patients with plasma glucose < 2.8 mmol/L (< 50 mg/dL) in the first 48 h of life and < 3.3 mmol/L (< 60 mg/dL) beyond 48 h of life [19–21].

Both of our patients presented with significant hypoglycemia during their first few days of life that persisted up to 2 weeks of age. Hyperinsulinism was evident in each of these patients based on their biochemical evaluation during hypoglycemia. Mild liver dysfunction was documented in both patients which was not severe enough to account for such low glucose levels while receiving adequate alimentary nutrition.

Our first patient was diagnosed with transient tyrosinemia of infancy based on her biochemical evaluation. Based on our review of the literature, there has been no previous report of hyperinsulinism in infants with transient tyrosinemia, and although hypoglycemia has been described in patients with HT1, the mechanism of this presentation is not well understood [1, 3].

There are various etiologies of congenital hyperinsulinism, which include eleven monogenic forms as well as milder episodes as seen in infants with transient perinatal stress and prematurity [18]. Different amino acids have been known to induce positive as well as negative effects on beta cell insulin secretion [12].

Leucine is an amino acid whose well known insulinotropic effects are mediated by several different mechanisms, including the production of alpha ketoglutarate and allosteric activation of glutamate dehydrogenase (GDH) [12]. Patients with leucine sensitive hypoglycemia were described as early as in 1960, although the mechanism was only understood after several decades [11]. Animal studies in sheep have demonstrated a rise in insulin secretion with intravenous administration of amino acids such as leucine, glycine, alanine, lysine and serine. Similarly, phenylalanine, a precursor to tyrosine, has been shown to induce insulin secretion in sheep although no such effects have been demonstrated with tyrosine [14].

Human studies have also demonstrated this insulinotropic effect of amino acids, both individually and in amino acid mixtures [8]. In an experiment in healthy humans, Fajans et al. elicited insulin release following IV infusion of certain amino acids. Infusion with arginine, lysine, leucine, phenylalanine, valine, methionine and histidine all resulted in insulin production with arginine being maximally potent and histidine the least [8].

Our first patient with transient tyrosinemia had transiently elevated levels of leucine, phenylalanine, isoleucine and valine (Table 2). The patient with HT1 had transient elevation of alanine, valine and lysine which normalized by two months of life. Both patients demonstrated elevated tyrosine levels (Table 2). We postulate that these elevated amino acids had transient insulinotropic effects, therefore manifesting as hypoglycemia in both patients.

**Table 2:** Amino acid levels in both infants

Amino acid levels μM (normal values)	Patient I			Patient II		
	Day 19 of life	Day 22 of life	Approximately 2 months of life	Day 7–8 of life	Day 12 of life 3 days after starting Nitisinone	17 months of life
Leucine 47–155	⇑ *164.1*	⇑ *166.7*	78.3	126.1	157.7	103.1
Alanine 131–710	534.8	526.6	352.7	⇑ *841.3*	628.6	302.3
Isoleucine 31–86	⇑ *92.9*	⇑ *102*	51.0	50.9	81.1	43.9
Valine 86–190	⇑ *258.3*	⇑ *234*	147.2	⇑ *265.7*	⇑ *290.5*	267.6
Lysine 52–196	⇑ *200.9*	⇑ *242.7*	*127.4*	⇑ *395.9*	⇑ *345.9*	69
Phenylalanine 31–75	⇑ *96.5*	⇑ *78.3*	41.3	53.6	12	72.6
Tyrosine 22–108	⇑ *904.3*	⇑ *660.5*	⇑ *211.8* *⊛ 79 (6 months of life)*	⇑ *506*	26.8	⇑ *426.4*

Values with upward arrow and in italic indicate high levels of amino acids

**Fig. 1:**
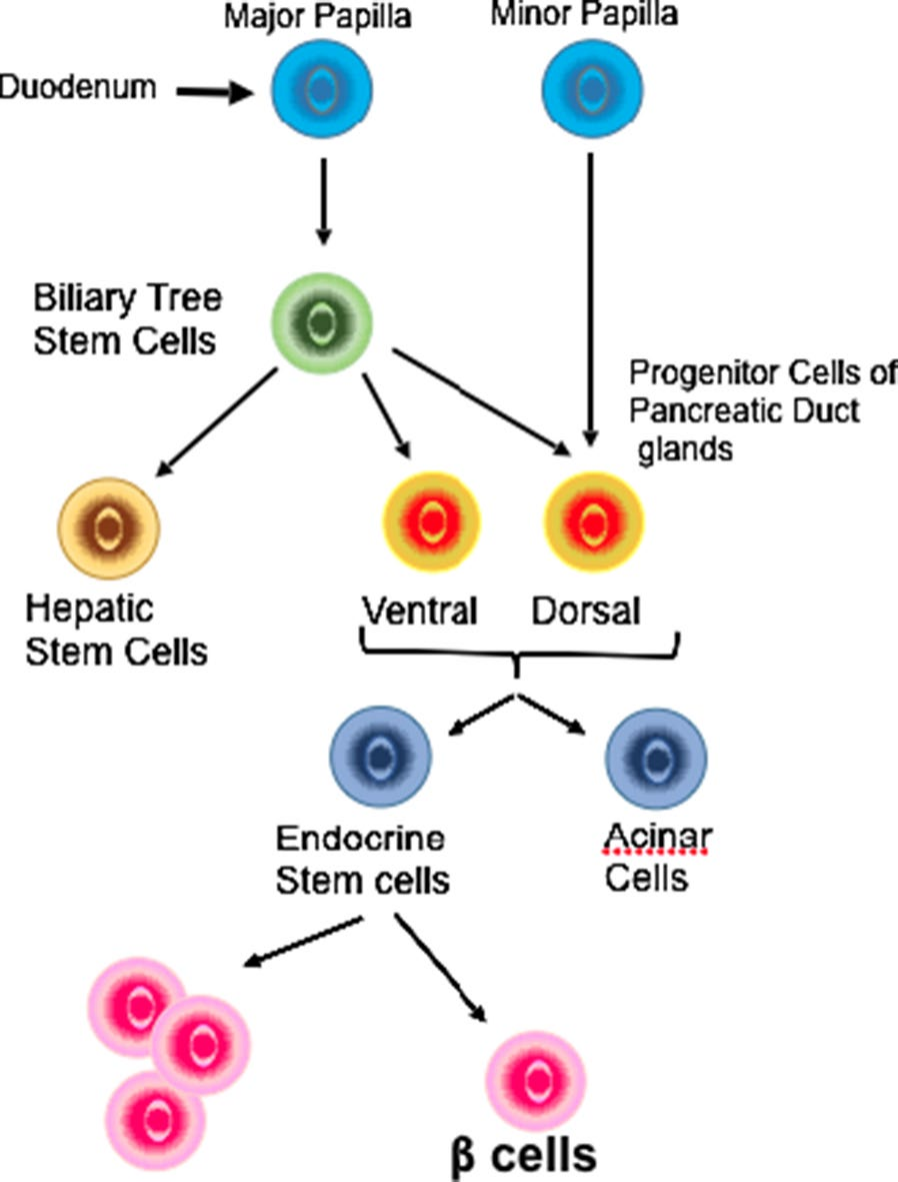
Common origin for the development of pancreatic β-cells form from bile duct epithelium. It could be postulated that abnormal development of liver cells or abnormalities within the biliary tree could affect the appropriate development and/or function of future pancreatic cells. The diagram is an adapted proposal illustrating a possible path to the development of pancreatic β-cells. Adapted from Cardinale et al. [12]

In addition, reports have demonstrated that patients with HT1 have hyperplasia of the islet cells of the pancreas [16, 17]. This anatomical change, therefore, could reflect and be responsible for the hyperinsulinism causing significant hypoglycemia. In HT1, there is constant damage to hepatic tissue by toxic metabolites. This results in liver repair and nodule formation by activation of the stem cell compartment. There is evidence that stem cells and progenitor cells in the major duodenal papilla give rise to the biliary tree stem cells, which eventually lead to organogenesis of both the liver and the pancreas [2]. Given the common origin of liver and pancreatic cells, one could propose that inadvertent activation of stem cells could lead to hyperplasia of pancreatic islet cells in addition to hepatocytes, leading to hyperinsulinism (Figure 1). This theory, however, could not explain hyperinsulinism in transient tyrosinemia where toxic metabolites do not accumulate.

We found one reported case series of three children with hyperinsulinemic hypoglycemia in the setting of HT1 that responded to diazoxide therapy [1]. All three patients required this medication only transiently, for 9, 18 and 34 months respectively. Our patient with HT1 required therapy until 17 months of life with some transient hypoglycemia during illness following discontinuation of medication. It is yet to be determined if his hypoglycemia will recur and require diazoxide again. However, hyperinsulinism in our patient with transient tyrosinemia was transient with discontinuation of therapy by 3.5 months of life.

## Conclusion

In conclusion, we report the first incidence hyperinsulinism in a patient with transient tyrosinemia of infancy. We also report a patient with HT1 diagnosed with hyperinsulinism requiring diazoxide therapy until 17 months of life. Both cases had transient, but prolonged hyperinsulinism that was diazoxide responsive. Although hyperinsulinemic hypoglycemia has been reported in patients with HT1, no clear mechanism for this has been delineated. We postulate that the elevation of certain amino acids such as leucine, glycine, valine, phenylalanine and isoleucine could lead to excess insulin production. This hypothesis has not been previously proposed. Although pancreatic hyperplasia has been described in HT1, its mechanism is not clear. It seems more likely that amino acids stimulating insulin production result in islet cell hyperplasia. The common origin of the liver and pancreas does not explain the etiology of hyperinsulinism in transient tyrosinemia of infancy and hence cannot be invoked in this scenario. Nevertheless, the major novelty of our report is that clinicians should be alert about potential hypoglycemia in a patient diagnosed with either transient or hereditary tyrosinemia type I. Fortunately, we show these patients to be diazoxide responsive, so that euglycemia can be achieved in a relatively safe and effective manner. This report provides evidence of the transient nature of the hypoglycemia along with guidance for treatment to the health care provider and reassurance to the parents. Our proposed theory of amino acid induced hyperinsulinism is a known entity but has not been previously described with these metabolic disorders. Further studies are necessary to delineate the precise mechanism in these infants.

## Data Availability

Data presented in this article is available in each of the patient’s electronic medical record.

## References

[1] Baumann U, Preece MA, Green A (2005). Hyperinsulinism in tyrosinaemia type I. J Inherit Metab Dis.

[2] Cardinale V, Wang Y, Carpino G (2012). The biliary tree—a reservoir of multipotent stem cells. Nat Rev Gastroenterol Hepatol.

[3] Chinsky JM, Singh R, Ficicioglu C (2017). Diagnosis and treatment of tyrosinemia type I: a US and Canadian consensus group review and recommendations. Genet Med.

[4] de Laet C, Dionisi-Vici C, Leonard JV (2013). Recommendations for the management of tyrosinaemia type 1. Orphanet J Rare Dis.

[5] de Leon DD, Stanley CA. Congenital hypoglycemia disorders: new aspects of etiology, diagnosis, treatment and outcomes: highlights of the proceedings of the congenital hypoglycemia disorders symposium, Philadelphia April 2016. Pediatr Diabetes. 2017;18(1):3–9. 10.1111/pedi.12453 PMC5473026 27753189

[6] de Lonlay P, Touati G, Robert J-J, Saudubray J-M (2002). Persistent hyperinsulinaemic hypoglycaemia. Semin Neonatol SN.

[7] Ferrara C, Patel P, Becker S (2016). Biomarkers of insulin for the diagnosis of hyperinsulinemic hypoglycemia in infants and children. J Pediatr.

[8] Floyd JC, Fajans SS, Conn JW (1966). Stimulation of insulin secretion by amino acids. J Clin Invest.

[9] Galcheva S, Demirbilek H, Al-Khawaga S, Hussain K (2019). The genetic and molecular mechanisms of congenital hyperinsulinism. Front Endocrinol.

[10] Ghosh A, Banerjee I, Morris AAM (2016). Recognition, assessment and management of hypoglycaemia in childhood. Arch Dis Child.

[11] Grumbach MM, Kaplan SL (1960). Amino acid and alpha-keto acid-induced hyperinsulinism in the leucine-sensitive type of infantile and childhood hypoglycemia. J Pediatr.

[12] Keane K, Newsholme P (2014) Metabolic Regulation of Insulin Secretion. In: Vitamins & Hormones. Elsevier, pp 1–33 10.1016/B978-0-12-800174-5.00001-6 24559912

[13] Kelly A, Tang R, Becker S, Stanley CA (2008). Poor specificity of low growth hormone and cortisol levels during fasting hypoglycemia for the diagnoses of growth hormone deficiency and adrenal insufficiency. Pediatrics.

[14] Kuhara T, Ikeda S, Ohneda A, Sasaki Y (1991). Effects of intravenous infusion of 17 amino acids on the secretion of GH, glucagon, and insulin in sheep. Am J Physiol-Endocrinol Metab.

[15] Mudd SH (2011). Hypermethioninemias of genetic and non-genetic origin: A review. Am J Med Genet C Semin Med Genet.

[16] Perry TL (1967). Tyrosinemia associated with hypermethioninemia and islet cell hyperplasia. Can Med Assoc J.

[17] Russo P, O’Regant S (1990). Visceral pathology of hereditary tyrosinemia type I. Am J Hum Genet.

[18] Stanley CA (2016). Perspective on the genetics and diagnosis of congenital hyperinsulinism disorders. J Clin Endocrinol Metab.

[19] Stomnaroska-Damcevski O, Petkovska E, Jancevska S, Danilovski D (2015). Neonatal hypoglycemia: a continuing debate in definition and management. Pril Makedon Akad Na Nauk Umet Oddelenie Za Med Nauki.

[20] Thompson-Branch A, Havranek T (2017). Neonatal hypoglycemia. Pediatr Rev.

[21] Thornton PS, Stanley CA, De Leon DD (2015). Recommendations from the pediatric endocrine society for evaluation and management of persistent hypoglycemia in neonates, infants, and children. J Pediatr.

[22] Transient Tyrosinemia of the newborn. https://www.orpha.net/consor/cgi-bin/OC_Exp.php?lng=en&Expert=3402.

